# The effect of acute and long-term physical activity on extracellular matrix and serglycin in human skeletal muscle

**DOI:** 10.14814/phy2.12473

**Published:** 2015-08-19

**Authors:** Marit Hjorth, Frode Norheim, Astri J Meen, Shirin Pourteymour, Sindre Lee, Torgeir Holen, Jørgen Jensen, Kåre I Birkeland, Vladimir N Martinov, Torgrim M Langleite, Kristin Eckardt, Christian A Drevon, Svein O Kolset

**Affiliations:** 1 Department of Nutrition, Institute of Basic Medical Sciences, Faculty of Medicine, University of OsloOslo, Norway; 2 Department of Physical Performance, Norwegian School of Sport SciencesOslo, Norway; 3 Department of Endocrinology, Morbid Obesity and Preventive Medicine, Oslo University Hospital and Institute of Clinical Medicine, University of OsloOslo, Norway

**Keywords:** Extracellular matrix, physical activity, proteoglycan, serglycin, serpin E1

## Abstract

Remodeling of extracellular matrix (ECM), including regulation of proteoglycans in skeletal muscle can be important for physiological adaptation to exercise. To investigate the effects of acute and long-term exercise on the expression of ECM-related genes and proteoglycans in particular, 26 middle-aged, sedentary men underwent a 12 weeks supervised endurance and strength training intervention and two acute, 45 min bicycle tests (70% VO_2_max), one at baseline and one after 12 weeks of training. Total gene expression in biopsies from *m. vastus lateralis* was measured with deep mRNA sequencing. After 45 min of bicycling approximately 550 gene transcripts were >50% upregulated. Of these, 28 genes (5%) were directly related to ECM. In response to long-term exercise of 12 weeks 289 genes exhibited enhanced expression (>50%) and 20% of them were ECM related. Further analyses of proteoglycan mRNA expression revealed that more than half of the proteoglycans expressed in muscle were significantly enhanced after 12 weeks intervention. The proteoglycan serglycin (SRGN) has not been studied in skeletal muscle and was one of few proteoglycans that showed increased expression after acute (2.2-fold, *P* < 0.001) as well as long-term exercise (1.4-fold, *P* < 0.001). Cultured, primary human skeletal muscle cells expressed and secreted SRGN. When the expression of *SRGN* was knocked down, the expression and secretion of serpin E1 (SERPINE1) increased. In conclusion, acute and especially long-term exercise promotes enhanced expression of several ECM components and proteoglycans. SRGN is a novel exercise-regulated proteoglycan in skeletal muscle with a potential role in exercise adaptation.

## Introduction

Approximately 640 individual skeletal muscles are found in the human body, comprising about 40% of body weight in lean individuals. Physical activity reduces the risk of numerous chronic diseases including type 2 diabetes, cardiovascular diseases, certain types of cancer, dementia, and sarcopenia (Colberg et al. 2010; American College of Sports Medicine, 2013). Both a single acute exercise bout and long-term exercise induce dramatic responses in skeletal muscle, including activation of signaling pathways, altered gene expression, metabolic adaptation (Egan and Zierath 2013), altered secretory activity (Trayhurn et al. 2011), and changes in muscle mass and functionality (Egan and Zierath 2013). These changes also include regulation and remodeling of the ECM (Kjaer 2004; Gustafsson 2011). The ECM is an important structural and functional part of muscle, making up a framework for attachment of contracting cells, as well as playing a role in proliferation, differentiation, migration, and polarization of cells (Hynes 2009). In skeletal muscle the ECM is highly structured and critical for force transmission and normal muscle function (Gillies and Lieber 2011; Lund and Cornelison 2013). Many muscle diseases and aging are associated with alterations in ECM (Serrano and Munoz-Canoves 2010; Lieber and Ward 2013).

The ECM contains components like collagens, elastin, glycoproteins, hyaluronic acid, and proteoglycans (Kjaer 2004; Hynes 2009; Gillies and Lieber 2011). Proteoglycans have been studied in relation to muscle cell differentiation as well as certain diseases (Brandan and Gutierrez 2013a,b). The most abundant proteoglycan in skeletal muscle is decorin (DCN) (Andrade and Brandan 1991; Melo and Brandan 1993; Velleman et al. 1997) but other proteoglycans are also expressed, such as syndecan and glypican family members found on cell surfaces and perlecan (HSPG2) found in basement membranes (Brandan and Gutierrez 2013b). Proteoglycans are located in the ECM and on cell surfaces where they are important for matrix assembly, cell attachment and as binding sites for growth factors. They have important roles in normal tissue remodeling and in processes leading to accumulation of ECM in hepatic and lung fibrosis, atherosclerosis, and diabetic kidney complications (Tabas et al. 2007; Baghy et al. 2012; Kolset et al. 2012). Proteoglycans are also located in intracellular secretory compartments (Kolset et al. 2004). They consist of core proteins covalently attached to one or several glycosaminoglycan (GAG) chains. These GAGs are sulfated and negatively charged and can therefore bind ligands through electrostatic interactions or more specific interactions involving structures with a defined sulfation pattern (Esko and Selleck 2002; Gandhi and Mancera 2008; Lindahl and Li 2009).

Norheim et al. (2011) have previously reported that many proteins secreted from cultured skeletal muscle cells are related to ECM and that most of them are regulated by physical activity. Moreover, many proteins are secreted and deposited in the ECM and involved in ECM turnover, including several proteases and protease inhibitors. The high level of ECM secretory products from muscle cells suggests that they have important functions in muscle adaptation to physical activity. However, it is surprising how little is known about the effect of physical activity on skeletal muscle ECM and proteoglycans. To our knowledge, no previous study has systematically investigated the effect of acute and long-term exercise on regulating skeletal muscle proteoglycans. In this study, we investigated the effects of acute and long-term exercise on the mRNA expression of ECM-related genes and proteoglycans in human skeletal muscle. Our data show that skeletal muscle express many proteoglycans and several of these are upregulated after a 45 min bicycle session and after 12 weeks of combined strength and endurance training.

In addition to the classical cell surface, basement membrane, and connective tissue proteoglycans, we report that SRGN is expressed in human skeletal muscle and is one of few proteoglycans to be upregulated after acute as well as long-term exercise. The proteoglycan SRGN has mostly been studied in hematopoietic cells (Kolset and Pejler 2011; Korpetinou et al. 2014). It consists of a small core protein (18 kDa) and several GAG chains that are attached to the central part of the core protein. SRGN is located in secretory vesicles or granules where it is involved in regulating storage or secretion of histamine, serotonin, and some secretory proteins (Abrink et al. 2004; Kolset and Pejler 2011; Korpetinou et al. 2014). The extracellular functions of SRGN have not been studied to any great extent. However, it has been observed on the surface of multiple myeloma cells bound to CD44 (Purushothaman and Toole 2014) and shown to be involved in cell adhesion (Skliris et al. 2013; Purushothaman and Toole 2014), tumor growth and vascularization (Purushothaman and Toole 2014).

SERPINE1 is an exercise-regulated (Norheim et al. 2011), antifibrinolytic protease inhibitor that inhibits tissue plasminogen activator and urokinase, the two major activators of plasminogen (Gramling and Church 2010; Ghosh and Vaughan 2012). The plasminogen activation system is important for ECM remodeling in skeletal muscle (Serrano and Munoz-Canoves 2010) and has been related to exercise-induced adaptation and muscle regeneration (Hittel et al. 2003; Koh et al. 2005). Furthermore, SERPINE1 has been implicated in fibrosis (Ghosh and Vaughan 2012) and identified as an antiangiogenic factor (Gramling and Church 2010). Here, we report a potential role for SRGN in the regulation and secretion of SERPINE1.

## Materials and Methods

### Human training intervention

**Figure 1:**
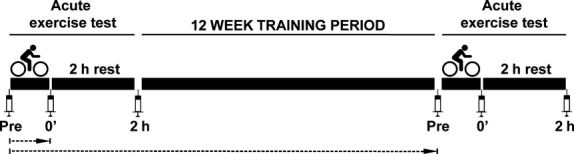
Design of the MyoGlu intervention study. Twenty-six middle-aged, sedentary men underwent a 12 weeks supervised training intervention. Before and after the intervention the participants undertook a 45 min acute bicycle test (70% VO_2_max). Muscle biopsies and blood samples were harvested before (Pre), immediately after (0′) and 2 h after the end of the bicycle sessions (2 h). Adipose tissue biopsies were collected 30–60 min after the two bicycle tests. To study the effect of an acute exercise bout, the main comparison made was between the samples taken before and immediately after 45 min bicycling at baseline of the study, indicated with the short dashed arrow. The effect of 45 min bicycling after the 12 weeks intervention was also investigated and the results were similar to the baseline data. To study the effect of long-term training the samples harvested before the acute tests at baseline and after 12 weeks were compared, indicated by the long dashed arrow.

A training intervention study was performed as described elsewhere (Li et al. 2014; Norheim et al. 2014) (Fig.1). The study was approved by the National Committee for Research Ethics North, Tromsø, Oslo, Norway (NCT01803568) and adhered to the standards set by the Declaration of Helsinki. All participants gave written informed consent.

Sedentary men with less than 1 bout of exercise per week the previous year and an age of 40–65 years were recruited. The participants (*n* = 26) had an average BMI of 26.2 kg/m^2^ (min 20.6, max 32.2). They were initially recruited to a control group (*n* = 13) with normal glucose metabolism (BMI 25.5 ± 2 kg/m^2^) and a dysglycemic group (*n* = 11) with impaired fasting glucose ≥5.6 mmol/L and/or 2 h serum glucose ≥7.8 mmol/L in an oral glucose tolerance test (BMI 29 ± 2.4 kg/m^2^). Two subjects had an intermediate glucose tolerance and did not fulfill the inclusion criteria of any group. In this study, we did not investigate group differences and all analyses were performed with the two groups pooled and all subjects were included (*n* = 26).

The participants underwent 12 weeks of supervised training with two intervals bicycle sessions and two whole-body strength-training sessions per week. The strength training sessions included a 10 min aerobic warm-up and three sets of each of the following exercises: leg press, leg curl, chest press, cable pull-down, shoulder press, seated rowing, abdominal crunches, and back extension. The endurance training sessions included a session of 7 min intervals at 85% of maximum heart rate and a session of 2 min intervals at >90% of maximum heart rate. The number of 7 min intervals per session progressed from 3 to 4 after the second week and from 4 to 5 after 6 weeks. Number of 2 min intervals per session progressed from 6 to 7 after the second week and from 7 to 10 after 6 weeks. The participants had an attendance rate of 89%.

A 45 min bicycle test at 70% of VO_2_max was performed both before and after the 12 weeks training period. Participants refrained from strenuous physical activity 2 days before the test and had a standard endurance session 3 days before the last bicycle test. The bicycle tests were preceded and followed by muscle biopsies, adipose tissue biopsies, and blood sampling. Tests before the 12 weeks intervention included euglycemic hyperinsulinemic clamp, maximum strength, and body composition measured by magnetic resonance imaging (MRI)/MR spectrometry (MRS) (Li et al. 2014). All participants underwent a full body MRI examination and MRS within 3 weeks prior to the start of the training period and a new examination within 2 weeks of the final exercise test.

Blood samples and muscle biopsies from *m. vastus lateralis* were taken at 6 time points, before, directly after and 2 h after the acute bicycle tests (Fig.1). Subcutaneous fat biopsies were collected from the periumbilical region in a suction-based biopsy procedure at two time points; 30–60 min after the bicycle test, before and after 12 weeks of training intervention. Blood sampling was performed by standard antecubital venous puncture. Muscle tissue was obtained by needle biopsies. The biopsies before and directly after the bicycle sessions were taken in the same incision in the distal and proximal direction of *m. vastus lateralis*, so that the distance between biopsies was typically 3–8 cm. The biopsies collected after 2 h were taken from a second incision. Muscle biopsies were immediately rinsed in ice cold PBS and dissected free of blood vessels and fat before they were transferred to RNA-later (Qiagen, Hilden, Germany) over night and frozen at −80°C. Adipose tissue biopsies were immediately frozen in liquid nitrogen and stored at −80°C until further processing.

#### High throughput mRNA sequencing of mRNA from muscle and adipose tissue biopsies

To isolate mRNA, frozen muscle biopsies were crushed to powder with a pestle in a liquid nitrogen-cooled mortar. Muscle tissue powder and frozen adipose tissue biopsy pieces were homogenized using TissueRuptor (Qiagen) at full speed, twice for 15 sec in 1 mL QIAzol Lysis Reagent (Qiagen) and then total RNA was isolated using miRNeasy® Mini Kit (Qiagen) for muscle samples or RNeasy® Lipid Tissue Mini Kit (Qiagen) for adipose tissue samples. RNA integrity and concentration were determined using Agilent RNA 6000 Nano Chips on a Bioanalyzer 2100 (Agilent Technologies Inc, Waldbronn, Germany).

RNA from fat (200 ng) and muscle (500 ng) was reverse-transcribed into cDNA on a Gene Amp PCR 9700 thermal cycler with the high capacity cDNA reverse transcription kit (Applied Biosystems, Foster City, CA). The cDNA reaction mixture was diluted in water and an equivalent of 12.5 ng RNA was analyzed in each sample.

The cDNA samples generated from biopsies were deep-sequenced using the Illumina HiSeq 2000 system with multiplexed design (Norheim et al. 2014). The cDNA was sonically fragmented and size selected to 51 base pair long reads before amplification. On average the library size was 44.1 million single end reads with no difference between groups or time points. Read alignment was done against the UCSC hg19 annotated transcriptome and genome dated 14 May 2013. Tophat 2.0.8 with Bowtie 2.1.0 was used with default settings and allowing two mismatches per uniquely aligned read (Langmead et al. 2009; Kim et al. 2013). Aligned reads at any genomic location were visually inspected using the Integrative Genomics Viewer (Robinson et al. 2011).

EdgeR v3.4.2 (Robinson et al. 2010) was used for gene filtering strategies, normalization and testing for statistical significance (*P*-values) using a negative binominal generalized linear model in R v3.0.3 (The R Foundation, Vienna, Austria) following the edgeR developers’ instructions. Normalized gene expression levels are presented in Fragments Per Kilobase of transcript per Million mapped reads (FPKM). Correction for multiple testing (*q*-values) was performed using Benjamini–Hochbergs false discovery rate (FDR) control (Benjamini and Hochberg 1995), set at FDR < 10%.

Genes encoding ECM-related proteins were identified by using the MatrisomeDB (http://matrisomeproject.mit.edu/) developed by the Matrisome project (Naba et al. 2012). Genes encoding collagens, ECM glycoproteins, proteoglycans, ECM regulators, and affiliated proteins were included. Other secreted factors including growth factors were not included.

#### Immunohistochemistry on muscle biopsies

Human muscle biopsies were fixed in 4% paraformaldehyde (w/v), 0.025% glutaraldehyde (v/v) in PBS for 4–6 h at room temperature. The biopsies were stored in a 1:10 dilution of the fixative at 4°C until paraffin embedding and then cut into 5 *μ*m sections using a rotary microtome (HM 355 S; MICROM International GmbH, Walldorf, Germany) and mounted on Superfrost Plus glass slides (Thermo Scientific, Braunschweig, Germany). The sections were incubated at 37°C overnight and deparaffinized in xylene (Sigma-Aldrich, St Louis, MO) twice for 5 min. Then the sections were rehydrated stepwise in 100%, 96%, 70%, and 50% ethanol for 5 min each and rinsed in tap water for 5 min. Heat-induced antigen retrieval was done by heating in 1 mmol/L EDTA (pH 8) at 95°C for 15 min and cooling to room temperature for 20 min. Next, the muscle sections were incubated for 1 h in blocking solution 1 (PBS containing 0.05% Triton X-100, 1% BSA and 3% newborn calf serum) at room temperature, followed by overnight incubation at 4°C with primary antibody (anti-human SRGN, 1 *μ*g/mL, a kind gift from Professor Niels Borregaard, Rigshospitalet, Copenhagen, Denmark) diluted in blocking solution 2 (PBS containing 0.05% Triton X-100, 1% BSA and 10% newborn calf serum). Sections were incubated with fluorochrome-conjugated secondary antibodies (Alexa Flour 488, 5 *μ*g/mL; Invitrogen, Eugene, OR) in blocking solution 2. The slides were dried and mounted with ProLong Gold with DAPI (Invitrogen).

Images were obtained with an Olympus BX61 upright fluorescent microscope (Olympus, Tokyo, Japan) and a Zeiss LSM510 camera (Zeiss, Oberkochen, Germany) through a 40× or 63× oil objective lens, equipped with argon (488 nm), HeNe1 (543 nm) and diode (405) lasers. Pinhole was kept at 1 AU. The fluorochromes were excited sequentially.

### Cell culture studies

#### Cell culturing and electric pulse stimulation

Primary human myoblasts from either *m. obliquus internus abdominis* or *m. vastus lateralis* of five healthy, male donors (33–53 years of age) were isolated as previously described (Haugen et al. 2010). Myoblasts at passage 4 to 5 were proliferated in dishes coated with collagen type I from rat tail (Sigma-Aldrich), in DMEM/Ham’s F12 1:1 (Gibco, Life Technologies, Grand Island, NY; Sigma-Aldrich) containing GlutaMAX™ (Gibco, Life Technologies, Paisley, UK), 50 U/mL penicillin, 50 *μ*g/mL streptomycin, 5 mmol/L glucose, 10% fetal bovine serum, 1 nmol/L insulin, 10 ng/mL epidermal growth factor, 2 ng/mL basic fibroblast growth factor and 0.4 *μ*g/mL dexamethasone (Sigma-Aldrich). When the cultures were 80% confluent, the myoblasts were differentiated for 5 days to multinucleated myotubes by changing the medium to DMEM/Ham’s F12 1:1 (5 mmol/L glucose) containing GlutaMAX™, 50 U/mL penicillin, 50 *μ*g/mL streptomycin and 2% horse serum (Sigma-Aldrich). Some cell cultures were incubated with 1 *μ*g/mL lipopolysaccharide (LPS), 1 *μ*g/mL brefeldin A, 0–2 *μ*mol/L ionomycin or 1 *μ*mol/L forskolin (Sigma-Aldrich). After 5 days of differentiation the cultures were subjected to electric pulse stimulation (EPS) with a frequency of 1 Hz, pulse duration 2 ms and an intensity of 11.5 V for 24 h. EPS was applied in serum-free conditions with a C-Dish in combination with a C-Pace pulse generator (C-Pace 100; IonOptix, Milton, MA).

#### Serglycin gene silencing

To investigate whether silencing of *SRGN* mRNA expression can influence myoblast proliferation, human myoblast cultures of 50% confluence were incubated for 24 h with 10 nmol/L SRGN siRNA (Santa Cruz Biotechnology, Santa Cruz, CA) or scramble siRNA (Qiagen) and 8 *μ*L/mL HiPerfect Transfection Reagent (Qiagen) in normal proliferation medium. After another 48 h cell numbers were counted with a Countess™ automated cell counter (Invitrogen) and protein concentrations were measured with a Pierce™ BCA Protein Assay Kit (Thermo Scientific, Rockford, IL). The effect of *SRGN* silencing on the mRNA expression and secretion of SERPINE1 was investigated in myotubes. The muscle cell cultures were differentiated for 1 day and then incubated with 10 nmol/L SRGN siRNA or scramble siRNA and 16 *μ*L/mL HiPerfect Transfection Reagent in medium containing 2% horse serum for 24 h. After another 48 h the cells were incubated with serum-free medium for 24 h and the concentration of SERPINE1 in the conditioned medium was measured with a Quantikine ELISA Kit (RnD Systems, McKinley Place NE, MN). The silencing efficacy was quantified by reverse transcription polymerase chain reaction after 24, 48 and 96 h and untreated controls and controls incubated with only HiPerfect reagent were included.

#### Reverse transcription polymerase chain reaction

Total RNA was isolated from cell cultures with the RNeasy® Mini kit (Qiagen) according to the manufacturer’s protocol. RNA (550 ng) was reverse-transcribed into cDNA as described under the section on high throughput mRNA sequencing.

Predeveloped primers and probe sets (TaqMan assays; Applied Biosystems) were used to analyze mRNA levels of: large ribosomal protein P0 (*RPLP0*, Hs99999902_m1), myogenin (*MYOG*, Hs01072232_m1), myosin heavy chain 7 (*MYH7*, Hs01110632_m1), interleukin 6 (*IL6*, Hs00985639_m1), peroxisome proliferator-activated receptor gamma, coactivator 1 alpha (*PPARGC1A*, Hs01016719_m1) and *SRGN* (Hs01004159_m1). Relative target mRNA expression levels were calculated as 2^−[Ct(target)−Ct(RPLP0)]^.

#### Metabolic labeling of (^35^S)macromolecules

Human myoblasts or myotubes were incubated in 100 *μ*Ci/mL (^35^S)sulfate (Hartmann Analytic GmbH, Braunschweig, Germany) in standard medium for 24 h. Conditioned media were collected and centrifuged to remove cell debris and the cell fractions were lysed in Hepes buffer (pH 7.4) with 1% Triton X-100. To remove unincorporated (^35^S)sulfate and to recover (^35^S)sulfated macromolecules, medium and cell lysates were subjected to Sephadex™ G-50 Fine (GE Healthcare, Little Chalfont, UK) gel chromatography (Meen et al. 2011). The sulfated macromolecules were eluted in the void volume with 0.05 mol/L Tris–HCl, 0.05 mol/L NaCl, pH 8. The amount of (^35^S)macromolecules isolated was quantified by scintillation counting. Samples were concentrated using Amicon Ultra centrifugal tubes with a molecular mass cut off at 3 kDa and the protein concentrations were determined using the Pierce™ BCA Protein Assay Kit (Thermo Scientific).

#### SDS-PAGE

(^35^S)Macromolecules from cell and medium fractions were analyzed with SDS-PAGE. To degrade (^35^S)proteoglycans with (^35^S)chondroitin and dermatan sulfate chains, samples were incubated with 0.01 units of chondroitinase ABC (cABC; AMS Biotechnology, Abingdon, UK) in 0.05 mol/L Tris–HCl pH 8.0 containing 0.05 mol/L sodium acetate and 0.02% BSA for 37°C overnight. A unique feature of the proteoglycan SRGN is the resistance to degradation by trypsin (Stevens et al. 1985). To analyze the possible presence of trypsin-resistant (^35^S)proteoglycans in muscle cells cultures, samples were incubated with 0.08% trypsin-EDTA at 37°C overnight. Soybean trypsin inhibitor (Sigma-Aldrich, 0.625%, final concentration) was added the next day. For comparison on SDS-PAGE each sample contained the same amount of (^35^S)sulfate (18 000 mCi/well). To control for trypsin activity, dual color molecular weight standard was incubated with the same amount of trypsin-EDTA and trypsin inhibitor solutions. After SDS-PAGE, the 4–20% Criterion™ Precast gels (Bio-Rad, Hercules, CA) were incubated in fixative (isopropanol 25%, glacial acetic acid 10%) for 30 min and thereafter in Amersham Amplify Fluorographic Reagent (GE Healthcare) for 15 min. Dried gels were subjected to fluorography using Amersham Hyperfilm™ ECL (GE Healthcare) for approximately 4–5 days at −20°C.

#### Immunoprecipitation

Isolated (^35^S)macromolecules from media and cell fractions of myoblasts and myotubes incubated with or without LPS were subjected to Sephadex™ G-50 gel filtration. Samples containing equal amounts of protein were incubated with or without cABC or trypsin-EDTA (described above). Then the samples were incubated with 0.65 *μ*g/mL rabbit anti-human SRGN antibody (a kind gift from Professor Niels Borregaard, Rigshospitalet, Copenhagen, Denmark) overnight at 4°C and then with 30 *μ*g Dynabeads® Protein G (Invitrogen) for 1 h at 4°C. After three washing steps in buffer (0.05 mol/L Tris-HCl, 0.15 mol/L NaCl, 0.05% Triton X-100) followed by boiling for 5 min in Laemmli sample buffer, all samples were loaded on to a 4–20% SDS-PAGE gel, incubated in fixative and Amplify Fluorographic Reagent (GE Healthcare), dried and subjected to fluorography for 4–5 weeks at −20°C.

#### Immunohistochemistry of cultured muscle cells

Human muscle cells were cultured on glass slides coated with collagen type I from rat tail (Sigma-Aldrich), fixed in 4% paraformaldehyde for 10 min and washed in PBS and water. The fixed cells were incubated with affinity purified rabbit anti-human SRGN (1 *μ*g/mL) and monoclonal mouse anti-human desmin (10 *μ*g/mL; Abcam, Cambridge, UK), in PBS with 1.25% BSA and 0.2% saponin, overnight at 4°C. The cells were washed and incubated with relevant secondary antibodies (Alexa Flour 488 and 594 anti-IgG, 5 *μ*g/mL; Invitrogen) for 90 min at room temperature and washed in PBS and water. The slides were dried and mounted with ProLong Gold with DAPI (Invitrogen). Images were obtained with an Olympus BX61 as described for the muscle biopsies.

### Statistical analysis

Statistical analysis of in vitro data was performed using SPSS 20.0 software (IBM Corporation, Armonk, NY) and Microsoft Excel 2010. Effect measures are presented as mean ± standard error of the mean. To test for differences between conditions or time points we used ANOVA and/or Student’s *t-*test.

## Results

### ECM-related genes are regulated by acute exercise in skeletal muscle

We used global mRNA sequencing to monitor the total mRNA expression in biopsies from *m. vastus lateralis* from 26 men. Immediately after a 45 min bicycle session at baseline of the 12 weeks training intervention (Fig.1), about 550 gene transcripts were significantly and more than 50% upregulated (FDR < 0.05). Of these, 28 genes (5%) encoded structural or regulatory components of the ECM (Table1). Many of the genes with increased expression after acute exercise were proteases or protease-inhibitors involved in turnover of ECM. Six genes in the serine protease inhibitor family (serpins) were upregulated. The metallopeptidase *ADAMTS4* was by far the most upregulated ECM-related gene (18.9-fold). The proteoglycan syndecan 4 (*SDC4*) had a high expression level (16.5 FPKM) and increased 1.9-fold during the acute bicycle session. Matricellular proteins are components of the ECM with a regulatory role. *CTGF* is encoding a matricellular protein that is an important growth factor regulating ECM biosynthesis (Kubota and Takigawa 2015) and was expressed at relatively high levels in the muscle biopsies (6.6 FPKM). Several members of the semaphorin (SEMA) family showed enhanced mRNA expression. These proteins are either secreted or membrane-bound and are related to several cellular functions such as cell adhesion, communication, and migration (Worzfeld and Offermanns 2014). A total of five ECM genes were more than 1.5 times downregulated, demonstrating that far fewer genes were downregulated than upregulated.

**Table 1:** Extracellular matrix (ECM) related genes upregulated by acute exercise. Gene transcripts related to ECM that were more than 50% upregulated after an acute 45 min exercise session in *m. vastus lateralis*

Gene symbol	FPKM*	Fold change†	*P*-value	*q*-value‡
ADAMTS4	0.4	18.9	6E-67	3E-64
THBS1	0.4	8.6	2E-22	6E-21
CYR61	9.3	8.0	8E-40	9E-38
ADAMTS1	5.9	7.2	1E-85	1E-82
SERPINE1	0.8	4.8	1E-27	5E-26
PLAU	2.6	3.6	7E-21	2E-19
TNFAIP6	0.5	3.2	3E-14	7E-13
CTGF	6.6	3.1	1E-27	6E-26
SERPINH1	6.7	3.0	1E-43	2E-41
SEMA4C	3.1	2.7	1E-80	1E-77
MMP19	0.3	2.2	3E-06	2E-05
SRGN	7.8	2.2	4E-53	1E-50
SERPINB9	0.8	2.2	2E-26	1E-24
SERPINA1	0.2	1.9	4E-06	3E-05
SDC4	16.5	1.9	1E-28	8E-27
SEMA3F	2.0	1.9	4E-45	6E-43
SEMA6B	1.6	1.9	1E-34	1E-32
CRISPLD2	3.3	1.8	4E-25	2E-23
HAPLN3	0.9	1.8	2E-11	2E-10
SERPINB8	0.3	1.8	3E-16	8E-15
SERPINA3	0.5	1.7	5E-05	3E-04
SEMA7A	0.6	1.7	5E-17	1E-15
CLEC1A	1.0	1.7	5E-08	5E-07
TNC	0.4	1.6	2E-05	1E-04
VWA1	1.5	1.6	5E-21	2E-19
ADAM8	0.3	1.5	3E-06	2E-05
TIMP1	6.8	1.5	1E-10	2E-09
POSTN	0.5	1.5	4E-04	2E-03

* Gene expression level at baseline measured by mRNA sequencing, expressed as Fragments Per Kilobase of transcript per Million mapped reads (FPKM).

† The expression just after 45 min bicycling as compared to before, at baseline of the 12 weeks intervention.

‡ False discovery rate.

**Figure 2:**
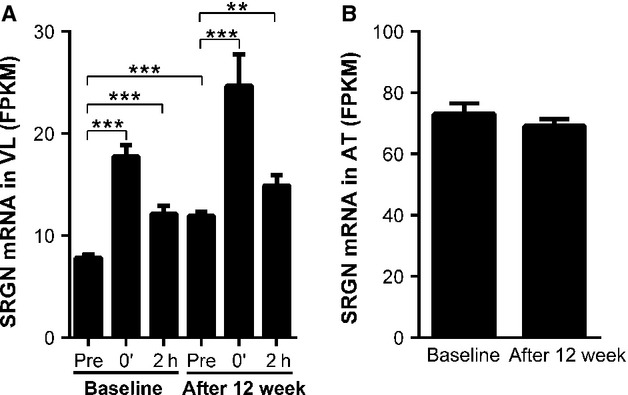
Serglycin mRNA is upregulated in *m. vastus lateralis* after acute and long-term training. Expression of *SRGN* mRNA in: (A) muscle biopsies collected before (Pre); immediately after 45 min ergometer cycling (70% VO_2_max) (0′); and after 2 h rest (2 h); before and after 12 weeks of training; (B) adipose tissue before and after long-term training. Data are expressed as FPKM. Bars depict means ± SEM. **P < 0.01, ***P < 0.001.

Because proteoglycans are important constituents of ECM and have not been systematically investigated in response to training, we decided to investigate a broad specter of them in further detail. Of the 39 investigated proteoglycans, 23 were consistently expressed in skeletal muscle and several were increased after acute exercise (Table2). The highest expression level was found for *DCN* (111 FPKM). High expression levels were also observed for the cell surface proteoglycans glypican-1 (*GPC1*) and *SDC4*. A novel finding in this study was the demonstration of *SRGN* expression in muscle biopsies (7.8 FPKM), which also had the highest induction after 45 min bicycling, both before (2.2-fold) and after 12 weeks of training intervention (1.9-fold; Fig.2A). Before the intervention all 26 participants showed an acute increase in *SRGN* mRNA (range 1.3–3.9-fold). Furthermore, *SDC4* mRNA was increased 1.9-fold (Table2) and *CD44* and chondroitin sulfate proteoglycan 4 (*CSPG4*) were also significantly upregulated immediately after the 45 min acute work-load. In contrast, asporin (*ASPN*), agrin (*AGRN)*, and leprecan (*LEPRE1*) mRNA levels were significantly reduced.

**Table 2:** Proteoglycans expressed in *m. vastus lateralis* and the mRNA regulation after an acute exercise session

Gene	Gene symbol	FPKM*	Fold change†	*P*-value	*q*-value‡
Serglycin	SRGN	7.8	2.2	4E-53	1E-50
Syndecan 4	SDC4	16.5	1.9	1E-28	8E-27
CD44 antigen	CD44	1.8	1.2	3E-10	3E-09
Chondroitin sulfate proteoglycan 4	CSPG4	2.6	1.2	2E-04	9E-04
Versican	VCAN	0.6	1.1	1E-02	4E-02
Syndecan 2	SDC2	4.6	1.1	1E-01	2E-01
Decorin	DCN	110.9	1.1	2E-01	3E-01
Biglycan	BGN	2.9	1.1	5E-01	6E-01
Syndecan 3	SDC3	2.6	1.0	4E-01	5E-01
Glypican 1	GPC1	43.8	1.0	6E-01	7E-01
Lumican	LUM	10.0	1.0	9E-01	9E-01
Perlecan	HSPG2	12.4	1.0	9E-01	9E-01
Collagen, type XVIII, alpha 1	COL18A1	5.8	1.0	9E-01	9E-01
Testican 2	SPOCK2	1.7	1.0	3E-01	5E-01
Podocan-like 1	PODNL1	0.5	0.9	4E-01	5E-01
Osteoglycin	OGN	0.6	0.9	7E-01	8E-01
Podocan	PODN	5.6	0.9	1E-01	2E-01
Testican 1	SPOCK1	1.9	0.9	2E-01	3E-01
Glypican 4	GPC4	8.2	0.9	3E-05	2E-04
Agrin	AGRN	1.9	0.8	2E-05	1E-04
Glypican 3	GPC3	0.5	0.8	1E-01	3E-01
Asporin	ASPN	2.5	0.8	6E-08	5E-07
Leprecan	LEPRE1	1.9	0.7	5E-05	3E-04

Sixteen other proteoglycans were included in the analysis, but were not expressed in skeletal muscle above the threshold: ACAN, BCAN, CSPG5, EPYC, GPC2, GPC5, GPC6, IMPG1, IMPG2, KERA, NCAN, NYX, OPTC, PRG2, PRG3, and SDC1.

* Gene expression level at baseline measured by mRNA sequencing, expressed as Fragments Per Kilobase of transcript per Million mapped reads (FPKM).

† The expression after 45 min bicycling as compared to before, at baseline of the 12 weeks intervention.

‡ False discovery rate.

### ECM genes are regulated by long-term exercise

The skeletal muscle was affected by 12 weeks of combined endurance and strength training; thigh muscle area (measured by MRI) increased 7.5% (*P* < 0.001), and muscle strength (measured by a maximum leg press test) increased 9.6% (*P* < 0.001) (Table3). A substantially larger number of ECM-related transcripts were enhanced during 12 weeks of training than after acute exercise. In total, 280 genes were significantly upregulated more than 50% (FDR < 0.05) in *m. vastus lateralis* after the long-term exercise and 20% of these were related to ECM (Table4). The matricellular protein secreted protein acidic and rich in cysteine (*SPARC*) had the highest expression level (71.5 FPKM) of all the upregulated, ECM-related genes and increased 76% during 12 weeks of training. Matrix-remodeling-associated protein 5 (*MXRA5*) had the highest relative increase of 2.8-fold although the absolute expression level was low (0.5 FPKM). Many mRNAs of structural ECM components were enhanced, including 10 collagens (*COL*), elastin (*ELN*), laminins (*LAMA4*, *LAMB1*, *LAMC3*), and nidogen 1 and 2 (*NID1*, *NID2*). Five members of the *ADAMTS* family and two *SERPIN*s were also upregulated. Only two ECM-related genes were more than 1.5-fold downregulated; laminin beta 3 (*LAMB3*; FC = −1.56, *P* < 0.001) and chondroadherin (*CHAD*; FC = −1.52, *P* < 0.001).

**Table 3:** Characteristics of participants (*n* = 26) before and after 12 weeks training

	Baseline	12 weeks	Change (%)
Age (years)	51.2 (6.6)		
BMI (kg/m^2^)	26.3 (3.5)	26.1 (3.3)	−0.7 (3.4)
Thigh muscle area (cm^2^)	214 (55)	231 (56)	7.5 (4.8)*
Fat mass (L)	38.7 (9.5)	35.7 (8.4)	−8.2 (6.0)*
GIR (mg/kg/min)	5.9 (2.4)	7.9 (3.3)	23.5 (20.0)*
Leg strength (kg)	224 (41)	248 (45)	9.6 (6.4)*
VO_2_max (mL/kg/min)	40.6 (5.8)	45.8 (6.4)	11.2 (7.4)*

Values are mean (SD).

* Significant change after 12 weeks.

**Table 4:** Extracellular matrix (ECM)-related genes upregulated by 12 weeks of training. Gene transcripts related to the ECM that were more than 50% upregulated in *m. vastus lateralis* after 12 weeks of exercise

Gene symbol	FPKM*	Fold change†	*P*-value	*q*-value‡
MXRA5	0.5	2.8	4E-16	3E-14
COL1A1	4.9	2.4	5E-20	8E-18
COL3A1	16.7	2.4	3E-19	4E-17
COL4A1	14.1	2.4	2E-29	2E-26
THBS4	21.7	2.2	5E-22	1E-19
LOXL2	1.1	2.2	5E-27	3E-24
COL4A2	13.4	2.2	6E-28	4E-25
BGN	2.9	2.1	2E-17	2E-15
OGN	0.6	2.1	4E-08	6E-07
COL6A6	0.1	2.0	2E-08	3E-07
LOX	0.3	2.0	1E-25	4E-23
PXDN	3.0	2.0	4E-32	5E-29
WISP1	0.1	1.9	1E-11	3E-10
SEMA5B	0.2	1.9	9E-12	3E-10
ADAMTS7	0.5	1.8	5E-21	9E-19
ASPN	2.5	1.8	1E-17	1E-15
COL14A1	0.6	1.8	3E-11	7E-10
OMD	0.3	1.8	8E-07	8E-06
COLEC12	2.3	1.8	1E-20	2E-18
COL1A2	16.1	1.8	3E-12	1E-10
SPARC	71.5	1.8	1E-26	5E-24
CLEC10A	0.7	1.7	4E-07	4E-06
LAMB1	6.8	1.7	2E-18	3E-16
CTHRC1	1.3	1.7	5E-12	2E-10
PAMR1	0.9	1.7	1E-05	7E-05
LAMA4	3.0	1.7	3E-36	1E-32
NID2	2.3	1.7	9E-25	3E-22
VWA1	1.5	1.7	9E-18	1E-15
LAMC3	0.1	1.7	2E-04	1E-03
KCP	0.2	1.7	6E-09	1E-07
ITIH3	0.5	1.7	1E-05	9E-05
IGFBP3	3.5	1.7	5E-18	5E-16
CLEC7A	0.6	1.6	4E-14	2E-12
AGRN	1.9	1.6	5E-21	9E-19
ADAMTS15	0.8	1.6	1E-22	3E-20
KAZALD1	2.1	1.6	1E-15	7E-14
EMILIN3	0.4	1.6	3E-04	1E-03
COL5A2	2.9	1.6	6E-13	2E-11
PLXNB3	0.1	1.6	1E-05	9E-05
ECM2	1.9	1.6	4E-17	4E-15
THBS1	0.4	1.6	3E-06	2E-05
ADAMTS8	0.3	1.6	3E-04	1E-03
SERPINE1	0.8	1.6	7E-07	7E-06
ADAMTSL3	0.8	1.6	4E-15	2E-13
NID1	4.3	1.6	4E-18	4E-16
ADAMTS2	0.3	1.6	1E-06	1E-05
IGFBP2	4.0	1.6	2E-11	5E-10
SERPINH1	6.7	1.5	3E-20	4E-18
HSPG2	12.4	1.5	1E-21	3E-19
LOXL3	0.4	1.5	7E-08	9E-07
MMP14	2.7	1.5	2E-15	1E-13
IGSF10	0.1	1.5	2E-04	8E-04
GREM1	0.7	1.5	8E-08	1E-06
CLEC11A	1.4	1.5	9E-08	1E-06
AEBP1	2.1	1.5	1E-08	2E-07
COL15A1	21.0	1.5	6E-13	2E-11
ELN	2.4	1.5	7E-09	1E-07
COL5A1	2.7	1.5	9E-11	2E-09
LUM	10.0	1.5	2E-06	2E-05

* Gene expression level at baseline measured by mRNA sequencing, expressed as Fragments Per Kilobase of transcript per Million mapped reads (FPKM).

† The mRNA expression after 12 weeks of training as compared to baseline of the intervention.

‡ False discovery rate.

More than half of all the expressed proteoglycans were significantly increased after the 12 weeks training and six were upregulated more than 50% (Table5). Interestingly, the basement membrane proteoglycans *HSPG2*, *AGRN*, and *COL18A1* were all upregulated. Many of the small leucine-rich proteoglycans were also increased, including biglycan (*BGN*), osteoglycin (*OGN*), *ASPN* and lumican (*LUM*). Glypican 4 (*GPC4*) was the only proteoglycan to be significantly downregulated.

**Table 5:** Proteoglycans expressed in *m. vastus lateralis* and the mRNA regulation during long-term training

Gene	Gene symbol	FPKM*	Fold change†	*P*-value	*q*-value‡
Biglycan	BGN	2.9	2.1	2E-17	2E-15
Osteoglycin	OGN	0.6	2.1	4E-08	6E-07
Asporin	ASPN	2.5	1.8	1E-17	1E-15
Agrin	AGRN	1.9	1.6	5E-21	9E-19
Perlecan	HSPG2	12.4	1.5	1E-21	3E-19
Lumican	LUM	10.0	1.5	2E-06	2E-05
Collagen, type XVIII, alpha 1	COL18A1	5.8	1.5	2E-09	4E-08
Chondroitin sulfate proteoglycan 4	CSPG4	2.6	1.5	2E-08	3E-07
Serglycin	SRGN	7.8	1.4	5E-17	4E-15
Syndecan 3	SDC3	2.6	1.4	7E-18	7E-16
CD44 Antigen	CD44	1.8	1.3	8E-10	2E-08
Glypican 3	GPC3	0.5	1.3	1E-01	2E-01
Testican 1	SPOCK1	1.9	1.3	1E-04	7E-04
Versican	VCAN	0.6	1.2	4E-02	9E-02
Leprecan	LEPRE1	1.9	1.1	6E-04	3E-03
Testican 2	SPOCK2	1.7	1.1	9E-03	3E-02
Decorin	DCN	110.9	1.0	6E-01	7E-01
Syndecan 2	SDC2	4.6	1.0	1E+00	1E+00
Podocan	PODN	5.6	0.9	4E-01	5E-01
Glypican 1	GPC1	43.8	0.9	1E-01	2E-01
Syndecan 4	SDC4	16.5	0.9	1E-01	2E-01
Podocan-like 1	PODNL1	0.5	0.9	2E-01	3E-01
Glypican 4	GPC4	8.2	0.8	1E-03	5E-03
Testican 3	SPOCK3	0.2	0.7	2E-01	3E-01

Sixteen other proteoglycans were included in the analysis, but were not expressed over the threshold: ACAN, BCAN, CSPG5, EPYC, GPC2, GPC5, GPC6, IMPG1, IMPG2, KERA, NCAN, NYX, OPTC, PRG2, PRG3, SDC1.

* Gene expression level at baseline measured by mRNA sequencing and expressed as Fragments Per Kilobase of transcript per Million mapped reads (FPKM).

† The mRNA expression after 12 weeks of training as compared to baseline of the intervention.

‡ False discovery rate.

*SRGN*, *CD44*, *CSPG4*, and versican (*VCAN*) were all increased by acute as well as long-term exercise and *SRGN* was the proteoglycan with the highest expression level of these. *SRGN* mRNA expression increased 40% during 12 weeks of training and an increase could be demonstrated in 24 of the 26 participants.

Biopsies from subcutaneous adipose tissue were also harvested before and after 12 weeks of training. In contrast to the skeletal muscle, total body fat measured by MRI was reduced 8.2% (*P* < 0.001). We observed a much smaller response to 12 weeks of training on gene expression levels in adipose tissue than in skeletal muscle. *ADAMTS4* was the only ECM-related gene upregulated more than 50%, whereas only collagen XXII*α*1 (*COL22A1)* was more than 1.5-fold decreased (−1.86-fold, *P* = 0.007). *SRGN* was expressed at high levels in adipose tissue, but the expression was unaffected by long-term exercise (Fig.2B).

### Intracellular localization of serglycin

**Figure 3:**
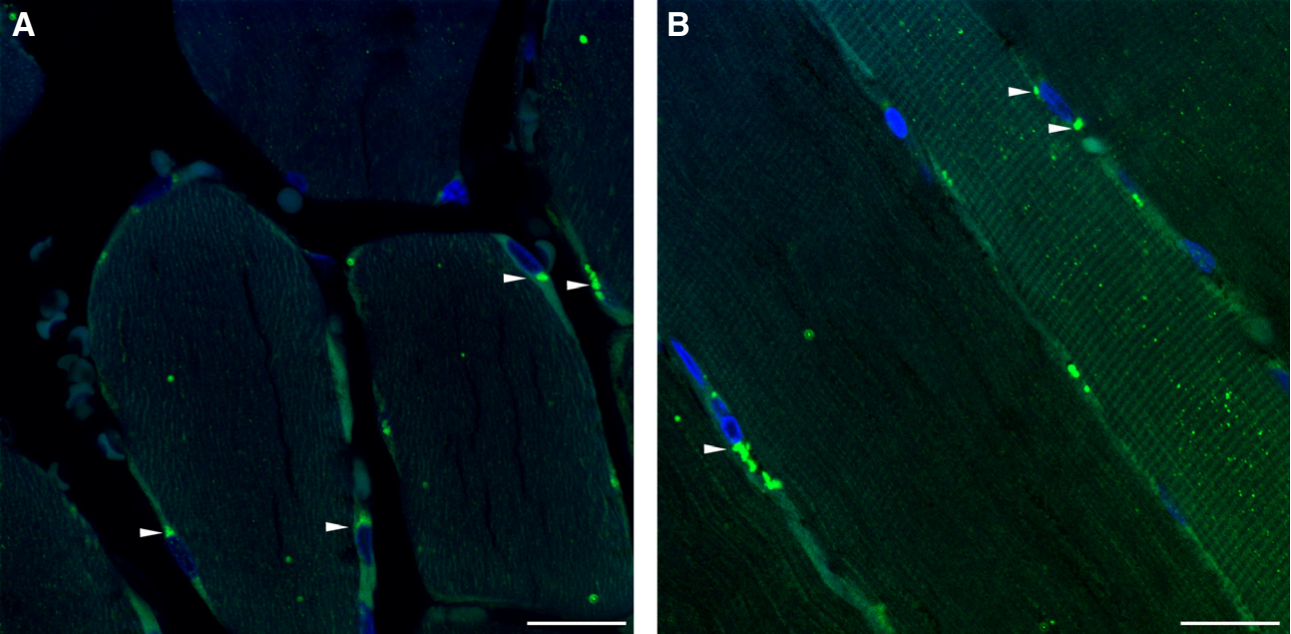
Intracellular localization of serglycin in human skeletal muscle. Fixed muscle sections from human *m. vastus lateralis* were stained with an antibody against SRGN (green) and DAPI nuclear stain (blue). (A) Cross section; (B) longitudinal section. SRGN-positive perinuclear staining is indicated by arrows. Scale bar represents 20 *μ*m. Staining was done on biopsies from six different donors taken at a single time point.

We chose to focus on SRGN in further studies because SRGN was one of few proteoglycans regulated by acute as well as long-term physical activity and it was expressed at a fairly high level and has previously not been studied in skeletal muscle. To evaluate the presence of SRGN in muscle tissue, biopsies from *m. vastus lateralis* were analyzed by immunostaining. SRGN was identified in the perinuclear region of muscle fibers (Fig.3).

**Figure 4:**
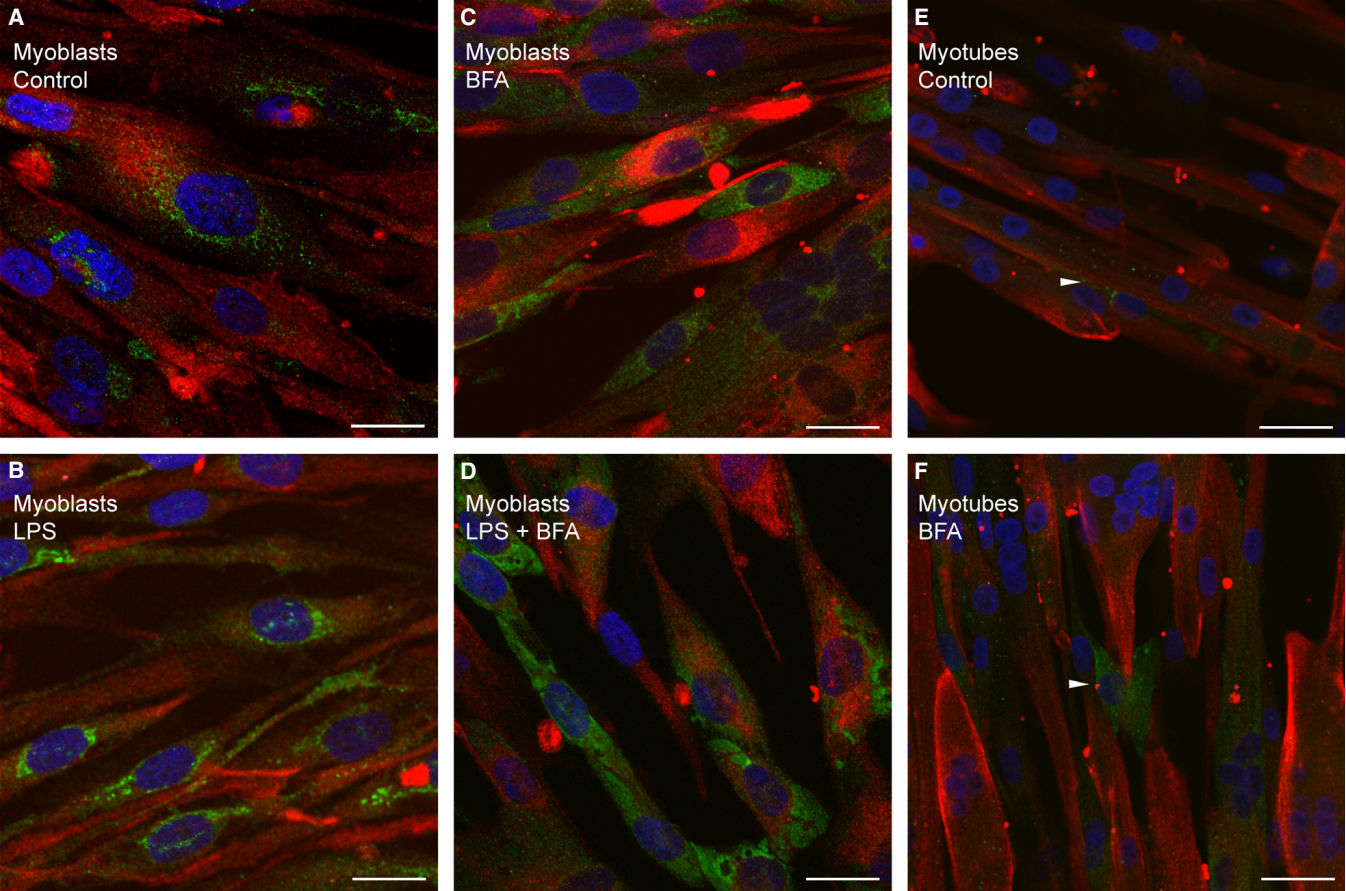
Perinuclear localization of serglycin in cultured muscle cells. Immunostaining of fixed cultured human muscle cells with antibodies against SRGN (green) and desmin (red) and DAPI nuclear stain (blue). Primary human myoblasts were incubated with: (A) vehicle, (B) LPS; (C) brefeldin A; or (D) both for 24 h. Primary human muscle cells were differentiated into multinuclear myotubes and incubated with: (E) vehicle or (F) brefeldin A for 24 h. Arrows indicate mononuclear cells. Scale bar represents 20 *μ*m in A–D and 32 *μ*m in E–F. The experiments were performed three times, with two different donors.

The presence of SRGN was also investigated in cultured, primary human muscle cells; both myoblasts (mononuclear cells) and myotubes (myoblasts that have fused and differentiated into multinuclear cells) were immunostained. SRGN was located in cytoplasmic vesicles and enriched in the perinuclear region of myoblasts. A much higher level of SRGN was detected in myoblasts (Fig.4A) as compared to myotubes (Fig.4E). In the differentiated cultures most cells were myotubes, although a few mononuclear cells were still present and these exhibited a higher SRGN signal as indicated by arrows in Figure4E and F. These data indicate that SRGN is predominantly expressed in myoblasts in vitro.

### In vitro expression and secretion of serglycin

**Figure 5:**
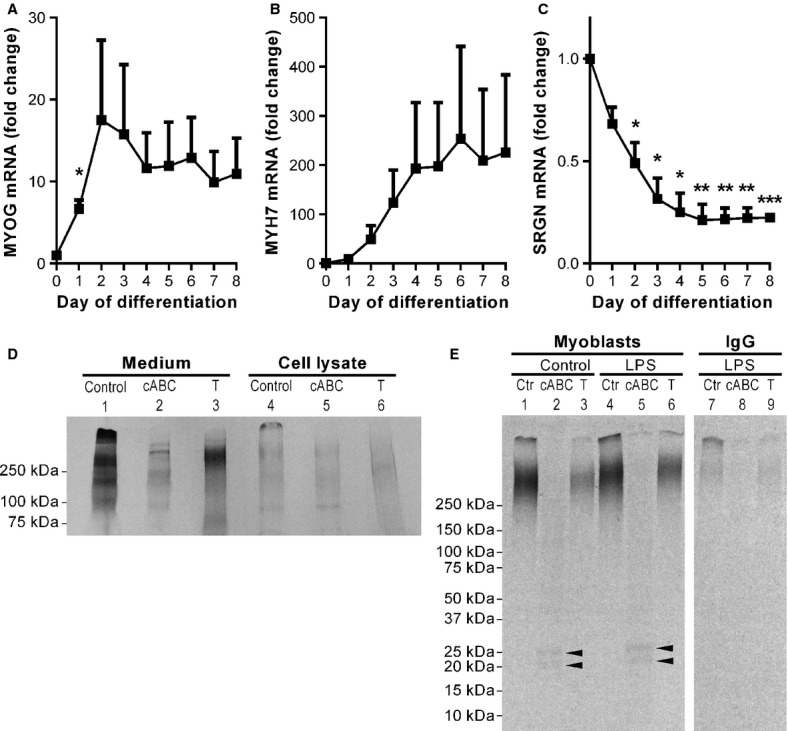
Serglycin is expressed and secreted from cultured human myoblasts. (A–C) Human myoblasts were differentiated to multinucleated myotubes for 8 days and mRNA was harvested every day during the differentiation. The mRNA expression of *MYOG*, *MYH7* and *SRGN* was determined by RT-PCR. Gene expression data are relative to the expression of *RPLP0* and the expression on day 0 and expressed as means of fold change. Error bars represent SEM. *P < 0.05, **P < 0.01, **P < 0.001, significant change from day 0. Experiments were performed with cells from three different donors. (D–E) Primary human myoblasts were incubated with (^35^S)sulfate for 24 h. (D) Conditioned medium (lane 1–3) and cell lysates (lane 4–6) were examined with SDS-PAGE and autoradiography. The samples were incubated with or without trypsin (T; lane 3 and 6) or cABC (lane 2 and 5). (E) Conditioned medium from human myoblasts incubated with (^35^S)sulfate and with or without 1 *μ*g/mL LPS (lane 1–6) for 24 h. SRGN was immunoprecipitated with anti-human SRGN antibody. IgG controls were included (lane 7–9). The samples were incubated with or without trypsin (T; lane 3, 6, 9) or cABC (lane 2, 5, 8). The experiment was performed three times, with two different donors.

To further evaluate the expression of *SRGN* in myoblasts and myotubes, the mRNA expression in differentiating muscle cell cultures was measured. During 8 days of differentiation the cells fused into multinuclear myotubes and the expression of myogenic genes such as *MYOG* and *MYH7* increased. The peak induction of *MYOG* was 10–36-fold and *MYH7* 18–623-fold, depending on cell donor (Fig.5A and B). In agreement with the immunocytochemistry data, the expression of *SRGN* gradually decreased during differentiation and was reduced 79% after 5 days (Fig.5C).

To study de novo synthesis and secretion of proteoglycans, primary human myoblasts were incubated with (^35^S)sulfate for 24 h. Radiolabeled sulfate is incorporated into the GAG chains of proteoglycans during biosynthesis. Cell and medium fractions were analyzed by SDS-PAGE and fluorography. SRGN consists of a small core protein with several GAG chains. Because SRGN and other proteoglycans have varying amounts of GAG chains they appear as broad bands after electrophoresis. Conditioned medium contained several (^35^S)proteoglycans. The major part was of molecular weight above 250 kDa (Fig.5D). Most of the radiolabeled material was susceptible to incubation with cABC (lane 2). cABC is an enzyme that only degrades GAG chains of the chondroitin (CS)/dermatan sulfate (DS) type. Hence, the major part of proteoglycans secreted from cultured primary muscle cells were CS/DS-proteoglycans. SRGN has been shown to be trypsin-resistant, because the core protein is protected by the densely packed GAG chains (Stevens et al. 1985). We made use of this criterion to investigate if human muscle express and secrete a trypsin-resistant proteoglycan. In lane 3 in Figure5D it is shown that a trypsin-resistant (^35^S)proteoglycan with molecular weight >250 kDa was present in the medium. The cell fractions were subjected to the same analyses and limited amounts of (^35^S)proteoglycan material were detected, supporting the notion that proteoglycans are mainly secretory products in cultured human muscle cells.

The trypsin-resistant band is likely to be SRGN. To investigate this further, conditioned medium from radiolabeled cells was subjected to immunoprecipitation with a SRGN antibody, followed by SDS-PAGE and fluorography (Fig.5E). A proteoglycan with a migration pattern similar to that observed for the trypsin-resistant band in Figure5D was recovered after immunoprecipitation. This band was also trypsin-resistant. In addition, after cABC incubation two core proteins with molecular weights of approximately 20 and 25 kDa appeared, most likely representing SRGN core proteins with different amounts of (^35^S)GAG stubs left after incubation with cABC. Two core proteins (20 and 35 kDa) appearing after cABC incubation of SRGN has also been demonstrated in monocytes (Kolset and Zernichow 2008). Our data confirm that primary human myoblasts secrete SRGN.

The amount of intracellular SRGN increased in myoblasts and myotubes incubated with brefeldin A. Brefeldin A is an inhibitor of the classical secretory pathway promoting secretory products to accumulate intracellularly (Kolset et al. 2002). Accumulation of SRGN was observed in the presence of brefeldin A (Fig.4C, D, and F), which is in agreement with the notion that SRGN is a secretory product. We could detect SRGN in multinuclear myotubes after incubation with brefeldin A, indicating that SRGN is secreted from myotubes as well as myoblasts.

### Regulation of serglycin in muscle cells

**Figure 6:**
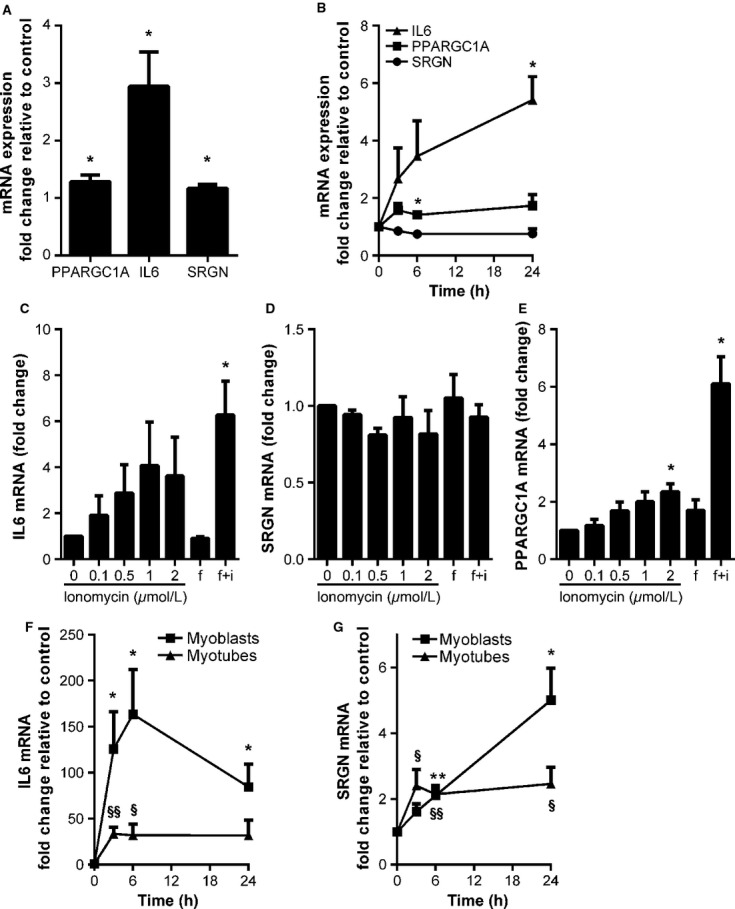
Electric pulse stimulation (EPS) and lipopolysaccharide (LPS) increases mRNA expression of serglycin. Differentiated human muscle cells were subjected to EPS (A) or incubated with: (B) 1 *μ*mol/L ionomycin for 3, 6, and 24 h; or (C–E) ionomycin (0–2 *μ*mol/L); forskolin (f; 1 *μ*mol/L); or both (f + i) for 3 h. (F–G) Human cultured myoblasts or myotubes differentiated for 5 days were incubated with 1 *μ*g/mL LPS for 3, 6, and 24 h. The mRNA expression of *PPARGC1A*, *IL6*, and *SRGN* was determined by RT-PCR and presented relative to the expression of *RPLP0* and the control (DMSO). Expressed as mean of fold change. *P < 0.05, **P < 0.01, significant difference from control in myoblasts. ^§^*P* < 0.05, ^§§^*P* < 0.01, significant difference from control in myotubes. Error bars represent SEM. All experiments were performed with cells from three to four different donors.

To gain more insight in the regulation of *SRGN* in human muscle cells, cultured myotubes were subjected to EPS to stimulate myotube contraction. EPS increased the expression of the exercise-responsive control genes *PPARGC1A* and *IL6* as well as *SRGN* (Fig.6A). The muscle cells were also incubated with ionomycin and/or forskolin to increase cytosolic calcium influx and cAMP levels. These agents have been shown to induce some of the changes observed in exercising skeletal muscle (Sparks et al. 2011). Incubation with ionomycin increased the expression of *PPARGC1A* and *IL6* (Fig.6B, C, and E). Forskolin alone did not influence gene expression, but in combination with ionomycin, the expression of *PPARGC1A* was upregulated 6.1-fold (*P* = 0.01) and *IL6* 6.3-fold (*P* = 0.04; Fig.6B and C). *SRGN* expression was not affected by ionomycin or forskolin (Fig.6D and E), suggesting that transcriptional regulation during exercise is mediated through other pathways than calcium and cAMP/PKA signaling.

In further studies on the regulation of *SRGN*, cultured myoblasts and myotubes were incubated with LPS. LPS is a ligand for toll-like receptor 4, has been shown to promote IL6 expression (Frost et al. 2006) and to be a potent modulator of SRGN expression in other cell types (Reine et al. 2014; Kolseth et al. 2015). We incubated muscle cells with LPS, to investigate whether SRGN is regulated in a similar manner in muscle cells as in other cell types. Incubation with LPS promoted increased *IL6* and *SRGN* mRNA in both myoblasts and myotubes (Fig.6E and F). The induction of *SRGN* was highest after 24 h incubation, with a five-fold increase in myoblasts and 2.5-fold increase in myotubes. Furthermore, conditioned medium immunoprecipitated with a SRGN antibody displayed a stronger trypsin-resistant band after LPS incubation (Fig.5E, lane 3 and 6), demonstrating increased secretion of SRGN. Finally, immunostaining of cultured myoblasts showed enhanced perinuclear and vesicular staining of SRGN after incubation with LPS (Fig.4B). When the cells were exposed to both LPS and brefeldin A, a very high staining intensity could be observed (Fig.4D).

### Serglycin is involved in the regulation of serpin E1

**Figure 7:**
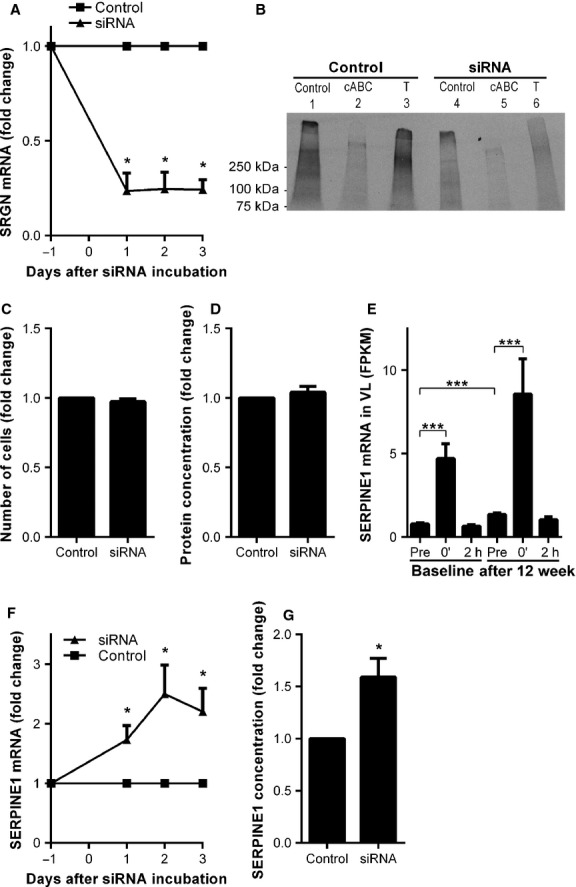
A potential role for serglycin in the regulation of serpin E1. The expression of *SRGN* was knocked down by incubating human primary muscle cells with siRNA. (A) The mRNA expression of *SRGN* in myotubes, 1, 2, and 3 days after end of incubation with siRNA. (B) Two days after transfection with siRNA, 24 h conditioned medium from myotubes was examined with SDS-PAGE and autoradiography. The samples were incubated with or without trypsin (T; lane 3 and 6) or cABC (lane 2 and 5). C-D) Myoblasts were incubated with SRGN siRNA and the number of cells (C) and protein concentration (D) were measured after 48 h. (E) Expression of *SERPINE1* mRNA in muscle biopsies collected before (Pre), immediately after 45 min of ergometer cycling (70% VO_2_max) (0′) and after 2 h rest (2 h) before as well as after 12 weeks of training. Data are expressed as FPKM. (F–G) The expression of SRGN was knocked down in human myotubes with siRNA and (F) *SERPINE1* was determined with RT-PCR 1, 2, and 3 days after end of transfection. (F) The concentration of SERPINE1 protein in medium conditioned from 48 to 96 h after transfection. Data on mRNA expression are relative to the expression of *RPLP0* and are presented as means of fold change. The experiments were performed 4–5 times (except for B, where *n* = 2). Bars depict means ± SEM. **P < 0.01, ***P < 0.001.

To investigate the functional role of SRGN in skeletal muscle cells, *SRGN* expression was knocked down by transfecting cultured muscle cells with siRNA. This led to a robust downregulation of *SRGN* mRNA (Fig.7A). As in previous experiments, de novo synthesis of proteoglycans was determined by (^35^S)sulfate labeling followed by SDS-PAGE. A weaker trypsin-resistant band was evident after incubation with siRNA (Fig.7B, lane 6). This band corresponds to SRGN (as demonstrated in Fig.5D and E).

In myoblasts *SRGN* was knocked down to examine whether it could influence proliferation rate. After 48 h there was no change in the cell number or protein concentration in cultures incubated with siRNA as compared to controls (Fig.7C–D).

SERPINE1 is a serine protease inhibitor and is a predicted functional partner of SRGN in the STRING database (Korpetinou et al. 2014; Szklarczyk et al. 2015). *SERPINE1* was one of the ECM-related genes that were upregulated on the mRNA level in skeletal muscle after both 45 min bicycling (4.8-fold, *P* < 0.001) and long-term training (1.6-fold, *P* < 0.001; Fig.7E). Interestingly, *SRGN* knockdown in differentiated muscle cells (>75%; Fig.7A) promoted a simultaneous increase in *SERPINE1* mRNA (Fig.7F) and SERPINE1 secretion (Fig.7G). After 48 h the secretion of SERPINE1 increased 60% (*P* = 0.048).

## Discussion

In this study, we demonstrate that many genes associated with ECM are regulated by acute as well as long-term exercise. After a 45 min exercise session 5% of all genes upregulated more than 1.5-fold were ECM related; many of these were proteases and protease inhibitors involved in turnover of ECM. After 12 weeks of combined endurance and strength training 20% of all upregulated genes were ECM related and these encoded proteases, protease inhibitors, structural components, and proteoglycans. Our lists of exercise-regulated ECM genes provide the basis for molecular follow-up studies, which may unravel some of the beneficial effects of ECM remodeling on health and skeletal muscle physiology.

The adipose tissue was influenced by the training intervention as fat mass was reduced by 8.2%. Furthermore, exercise is known to induce modulation of adipose tissue metabolism and secretory activity (Golbidi and Laher 2014). We monitored gene expression in adipose tissue and observed that only a few ECM-related genes were up- or downregulated. We only have biopsies from subcutaneous adipose tissue, thus we do not know whether other adipose tissue depots would exhibit different responses.

Skeletal muscle is a highly adaptable tissue. Adaptation to physical activity includes regulation of satellite cell activity, myogenesis and angiogenesis, tissue expansion and alterations in structural support (Yan et al. 2011; Egan and Zierath 2013; Blaauw and Reggiani 2014). Whereas the muscle undergoes hypertrophy in response to weight-bearing exercise, muscle wasting occurs when the muscle is less used as evident during extended inactivity. It is likely that ECM plays a major role in skeletal muscle adaptation to different physiological stimuli (Goody et al. 2015) and that ECM restructuring is necessary during muscle growth. Furthermore, ECM restructuring is important for angiogenesis. Degradation of basement membrane proteins by proteases has been shown to be necessary for activity-induced angiogenesis in rats (Haas et al. 2000).

It is surprising how little is known about the ECM in skeletal muscle. Our data show that a large proportion of the transcriptional response to physical activity in skeletal muscle is related to ECM components and enzymes involved in tissue remodeling. Fibroblasts are considered to be the major producers of ECM components in the muscle (Gillies and Lieber 2011), but muscle cells are also able to produce ECM proteins (Gillies and Lieber 2011; Norheim et al. 2011). Norheim *et al*. reported that many of the proteins secreted from human muscle cells in culture are related to the ECM and that they are regulated by strength training (Norheim et al. 2011). Both fibroblasts and muscle cells sense mechanical load during exercise. Mechanotransduction, partially through integrin receptors, plays a major role in regulating synthesis and turnover of ECM components (Kjaer 2004; Humphrey et al. 2014). Physical activity is accompanied by metabolic changes including improved insulin sensitivity and it is possible that this also can lead to altered expression of ECM-related genes. In murine skeletal muscle, insulin resistance has been associated with increased collagen content and reduced *MMP9* expression (Kang et al. 2011). Furthermore, insulin-resistant human subjects had an increased collagen content in skeletal muscle (Berria et al. 2006).

Also other studies have focused on the transcriptional response in skeletal muscle to physical activity. Timmons et al. (2005) used a microarray technique to analyze gene expression in muscle biopsies from 8 volunteers undergoing 6 weeks of endurance training. Gene ontology analysis revealed that the ECM gene families were most upregulated after training. It is also worth noting that most pathological conditions, insulin resistance, atrophy and aging in skeletal muscle are associated with alterations in the ECM (Lecker et al. 2004; Berria et al. 2006; Gillies and Lieber 2011; Kragstrup et al. 2011; Wood et al. 2014) and we suggest that exercise might counteract some of these pathological changes.

We chose to focus on proteoglycans, which to our knowledge have not been systematically investigated in skeletal muscle in relation to exercise. Of all the known proteoglycans expressed in muscle, more than half were upregulated after long-term training. *DCN* was the proteoglycan with the highest mRNA expression level. In our dataset *DCN* expression was not significantly changed by exercise, but it has previously been shown to be secreted from contracting skeletal muscle cells in vitro and to be involved in regulation of muscle hypertrophy by inhibition of atrophic pathways (Kanzleiter et al. 2014). Syndecans are transmembrane heparan sulfate proteoglycans and play a role in cell proliferation and fusion of satellite cells into differentiated myotubes (Brandan and Gutierrez 2013b). Both syndecan-3 and 4 are exclusively expressed in satellite cells in the muscle (Cornelison et al. 2001). In this study, we show that *SDC4* was upregulated after acute exercise, whereas syndecan-3 was upregulated after long-term exercise.

Serglycin has previously been regarded as a hematopoietic proteoglycan, but has also been identified in endothelial cells (Schick et al. 2001), chondrocytes (Zhang et al. 2010), adipocytes (Imoto-Tsubakimoto et al. 2013), and smooth muscle cells (Lemire et al. 2007). To our knowledge SRGN has not been studied in skeletal muscle before. Our data show that SRGN mRNA and protein are expressed in human muscle biopsies and cultured human muscle cells. In muscle biopsies, *SRGN* mRNA was upregulated after 45 min bicycling and after 12 weeks of combined endurance and strength training.

Electric pulse stimulation was used as an in vitro model of muscle contraction. When cultured muscle cells were activated with EPS, *SRGN* mRNA expression increased. However, *SRGN* expression was unaffected by pharmacological compounds increasing calcium and cAMP signaling, suggesting that the transcriptional regulation of *SRGN* is transmitted through other exercise-responsive signaling pathways. Furthermore, it has previously been shown that LPS and interleukin 1*β* signaling can induce synthesis and secretion of SRGN in hematopoietic (Kolseth et al. 2015) and endothelial cells (Reine et al. 2014). Here, we show that signaling pathways linked to LPS stimulation can be involved in regulation of *SRGN* expression in human muscle cells.

The functional role of SRGN in muscle cells remains unknown. Several of the documented functions of SRGN in other cell types are mediated through regulation of partner molecules with defined biological activities. SRGN is important for the formation of storage granules in mast cells, T lymphocytes and neutrophils where it participates in storage of several proteases (Abrink et al. 2004; Grujic et al. 2005; Niemann et al. 2007; Kolset and Pejler 2011; Wernersson and Pejler 2014) and has a role in secretion of matrix metalloproteinase-9 and urokinase in macrophages (Winberg et al. 2000; Pejler et al. 2003). SRGN also plays a role in storage of other secreted factors such as cytokines and growth factors (Kolset and Pejler 2011), including tumor necrosis factor *α* (Zernichow et al. 2006), chemokine (C-X-C motif) ligand 1 (CXCL1) (Meen et al. 2011), and interleukin 13 (Waern et al. 2012).

We speculate that the function of SRGN in muscle cells is similar to that in other cell types; to interact with secreted factors and regulate storage or secretion. The fact that SRGN is a predicted functional partner of SERPINE1 in the STRING database (Korpetinou et al. 2014; Szklarczyk et al. 2015) prompted us to investigate this relation in cultured muscle cells. We show that *SRGN* knockdown led to increased SERPINE1 expression and secretion. It remains to be investigated whether SRGN interacts with SERPINE1 and has a direct role in its regulation. Skeletal muscle is now considered an active endocrine organ that releases myokines, especially during physical activity (Eckardt et al. 2014; Catoire and Kersten 2015). Future studies will show whether other secreted factors in skeletal muscle cells are regulated by SRGN.

In addition to highlighting the importance of proteoglycans and ECM molecules, we hypothesize that SRGN is a molecule involved in storage and secretion of secreted factors in skeletal muscle and thereby involved in the adaptation of skeletal muscle due to physical activity.
